# Enhanced mirroring upon mutual gaze: multimodal evidence from TMS-assessed corticospinal excitability and the EEG mu rhythm

**DOI:** 10.1038/s41598-020-77508-x

**Published:** 2020-11-24

**Authors:** Jellina Prinsen, Kaat Alaerts

**Affiliations:** Neuromodulation Lab – Neurorehabilitation Research Group, Department of Rehabilitation Sciences, KU Leuven, Tervuursevest 101, Box 1501, 3001 Leuven, Belgium

**Keywords:** Neuroscience, Sensorimotor processing, Social neuroscience

## Abstract

Previous research has demonstrated that eye contact between actor and observer specifically enhances the ‘mirroring’ of others’ actions, as measured by transcranial magnetic stimulation (TMS)-induced motor evoked potentials (MEPs). However, it remains unknown whether other markers of mirror system activation, such as suppression of the EEG mu rhythm (8–13 Hz) over the sensorimotor strip, are also susceptible to perceived eye contact. Here, both TMS-induced MEPs and EEG mu suppression indices were assessed (in separate sessions) while 32 participants (mean age: 24y; 8m) observed a simple hand movement combined with direct or averted gaze from the actor. Both measures were significantly modulated by perceived eye gaze during action observation; showing an increase in MEP amplitude and an attenuation of the mu rhythm during direct vs. averted gaze. Importantly, while absolute MEP and mu suppression scores were not related, a significant association was identified between gaze-related changes in MEPs and mu suppression, indicating that both measures are similarly affected by the modulatory impact of gaze cues. Our results suggest that although the neural substrates underlying TMS-induced MEPs and the EEG mu rhythm may differ, both are sensitive to the social relevance of the observed actions, which might reflect a similar neural gating mechanism.

## Introduction

Ever since the discovery of ‘mirror neurons’ in the macaque brain^1^, firing not only when the monkey executes a motor action, but also when the monkey merely observes another individual performing that action, the description of a homologous action observation-execution matching or ‘mirror' system and its properties in humans has been a topic of increasing interest. While the exact role of the mirror system in human social cognition is still a matter of debate, it is generally assumed that the simulation of observed movements in the observer’s own motor system contributes to action recognition and understanding, including related socio-cognitive processes that are important for everyday social interactions such as imitation, mimicry, motor planning and gestural performance^2^. The mirror system has also been implicated to be involved in higher-order mentalizing processes, such as inferring others’ intentions (for a review, see ref.^3^; specific studies^4,5^), as well as empathy (a form of ‘emotional’ imitation^6^), but these notions are more controversial.

To identify patterns of human mirror system activity during movement observation, a variety of neuroimaging and electrophysiological techniques have been adopted. One commonly used method is transcranial magnetic stimulation (TMS), a non-invasive brain stimulation technique that activates cortical neurons via the administration of a brief magnetic pulse to the scalp. When TMS is administered over the somatotopically organized primary motor cortex (M1), it induces an involuntary muscle contraction or motor evoked potential (MEP) in the corresponding peripheral muscles (measured with electromyography; EMG), of which the peak-to-peak amplitude reflects variations in corticospinal excitability. In a seminal study, Fadiga et al. showed that TMS-evoked MEP amplitudes within the stimulated muscles are specifically enhanced during the observation of others’ movements compared to rest, presumably reflecting excitatory cortico-cortical connections between M1 and mirror regions in the brain^7^. Subsequent TMS studies have confirmed these observations (for a review, see ref.^8^), and provided evidence that the human observation-execution matching mechanism is specific to the muscles recruited in the observed actions^7,9–11^, with a close temporal coupling^12,13^.

Another commonly adopted method for investigating mirror system activity relates to the assessment of cortical rhythms in the electroencephalogram (EEG) or magnetoencephalogram (MEG). Of specific interest is the mu rhythm, which oscillates in the 8–13 Hz frequency band and is typically recorded over the sensorimotor regions of the brain (i.e. electrode positions C3, Cz, and C4 according to the 10–20 international system of electrode placement). It is maximally expressed during rest, when sensorimotor neurons fire in synchrony. When a person performs, observes or imagines themselves performing an action, the firing of these neurons becomes increasingly desynchronized, leading to a task-induced suppression of the mu rhythm^14–16^. The notion that decreased mu power is inversely related to sensorimotor activation received overall support from combined EEG-fMRI studies, showing a negative relationship between mu power and the BOLD signal in brain areas considered part of the mirror system^17–19^. Also several MEG studies, having superior spatial resolution compared to EEG, have shown that neural activity over sensorimotor cortices is significantly modulated by action observation and execution^20,21^. However, since the sensorimotor mu rhythm oscillates in the same 8–13 Hz frequency band and displays similar response properties as occipital alpha rhythms (i.e. dominant when at rest, suppressed by perceptual events and attentional processing), an important issue in EEG action observation studies is the potential contamination of the mu rhythm by changes in alpha^16,22^.

Albeit automatic, mirror system engagement upon action observation is not a static process, but has been shown to be highly adaptive and flexible depending on the social context in which the observed movements occur^23–25^. One highly powerful cue for driving interpersonal communication is eye contact. Whereas perceived direct gaze from others is indicative of their scrutiny and communicative intent, averted gaze cues signal that their attention is directed elsewhere. Accordingly, perceiving the gaze of others has been shown to influence various socio-cognitive processes and behavioral responses in the observer^26–28^. Also in terms of mirror system activity, several TMS studies have shown that under various experimental conditions, perceived communicative intent from the actor – as conveyed by different gaze cues – significantly modulates corticospinal excitability (i.e. MEPs) in the observer^29–32^. To date however it remains unexplored whether suppression of the EEG mu rhythm upon action observation is similarly modulated by social context or communicative intent, as conveyed by eye contact.

In this respect, it is worth noting that while both TMS and EEG techniques have been widely adopted to investigate observation-execution matching processes, the direct relationship between facilitation of corticospinal excitability as assessed with TMS and suppression of the EEG mu rhythm during action observation is not well established. The first study to investigate this matter recorded corticospinal excitability and mu suppression during movement observation, imagination and execution of simple hand actions in healthy adult participants^33^. The authors demonstrated that while both measures were significantly modulated by the experimental conditions designed to evoke mirror system activity (i.e. enhanced corticospinal excitability and increased mu suppression upon movement observation/imagination), changes in corticospinal excitability were not significantly correlated to changes in mu suppression at the inter-individual subject level^33^. Similarly, in two recent studies assessing observation-induced changes in corticospinal excitability and mu suppression, either during an action-related mentalizing task^34^ or during observation of goal-directed grasping movements^35^, no direct relationship was revealed between the two measures. Only one study to date has demonstrated a significant relationship between concurrent recordings of observation-induced mu suppression and corticospinal excitability in a mixed sample of schizophrenia patients and healthy controls, showing that enhanced MEP amplitudes were associated with increased mu suppression during the observation of videos depicting several types of biological movement (i.e. intransitive, transitive and interactive hand movements)^36^. However, when the participant groups were examined separately, none of the correlations remained significant.

Accordingly, it has been suggested that corticospinal excitability and mu suppression may represent different aspects of the mirror system, presumably due to the different spatial and temporal characteristics of the two techniques. While EEG mu suppression indexes the sum of post-synaptic neuronal activity over a large area (not restricted to M1) over a relatively long time period (typically > 1 s), TMS assesses changes in corticospinal excitability by stimulating a relatively small population of neurons (at the level of M1) at a discrete point in time. Moreover, TMS-induced MEP recordings of mirror system functioning are obtained at the level of the muscle (i.e. by means of EMG) and mainly reflect cortico*spinal* processes, compared to central cortical activity as assessed by EEG.

Within the present study, we adopted a multi-modal approach for assessing gaze-specific modulations of mirror system activity by recording TMS-induced MEPs and EEG mu rhythm suppression upon movement observation with variable communicative intent. In line with recent guidelines^16,22^, occipital alpha suppression during action observation was also taken into account. Key objectives were to assess whether suppression of the EEG mu rhythm is similarly susceptible to modulation by perceived communicative intent (i.e. the actor’s gaze direction); and whether eye contact-induced changes in corticospinal excitability are uniquely associated with changes in mu rhythm suppression. To do so, TMS and EEG indices were recorded in separate sessions while participants observed simple intransitive hand movements accompanied by either direct or averted gaze from the stimulus person. Based on previous findings in our lab^31^ and following recent recommendations^37,38^, a naturalistic two-person paradigm was adopted, incorporating a live stimulus person to convey the gaze and movement cues. In line with previous TMS studies demonstrating enhanced mirroring of others’ actions during eye-to-eye contact^29–31^, it was hypothesized that mirror system activation is enhanced during observation of movements accompanied with direct gaze from the actor, compared to movements accompanied with averted gaze from the actor.

## Method and Materials

### Participants

A total of 32 individuals (20 men and 12 women) aged between 18 and 36 years old (mean ± SD: 22;9 ± 3;7 years; months) participated in this study. All participants were right-handed, which was confirmed with the Edinburgh Handedness Questionnaire (EHQ)^39^. Exclusion criteria comprised medication use, any diagnosed psychiatric (e.g. ASD, ADHD) or neurological disorder (e.g. stroke, epilepsy, concussion), left handedness or any contraindication for TMS^40^. Written informed consent was obtained from all participants prior to the experimental procedure. Ethical approval for the experimental protocol was granted by the local Ethics Committee for Biomedical Research at the University of Leuven in accordance to the Declaration of Helsinki^41^.

### Experimental protocol and stimuli

Mirror system activity was investigated in two assessment sessions conducted on the same day, with a fifteen minute break between sessions. In one session, stimulus presentation was accompanied with transcranial magnetic stimulation (TMS) in order to assess excitability at the level of the primary motor cortex (M1). In the other session, electroencephalography (EEG) assessments were performed in order to measure mu rhythm suppression. The order of assessment method (TMS or EEG) was counterbalanced across participants.

**Figure 1:**
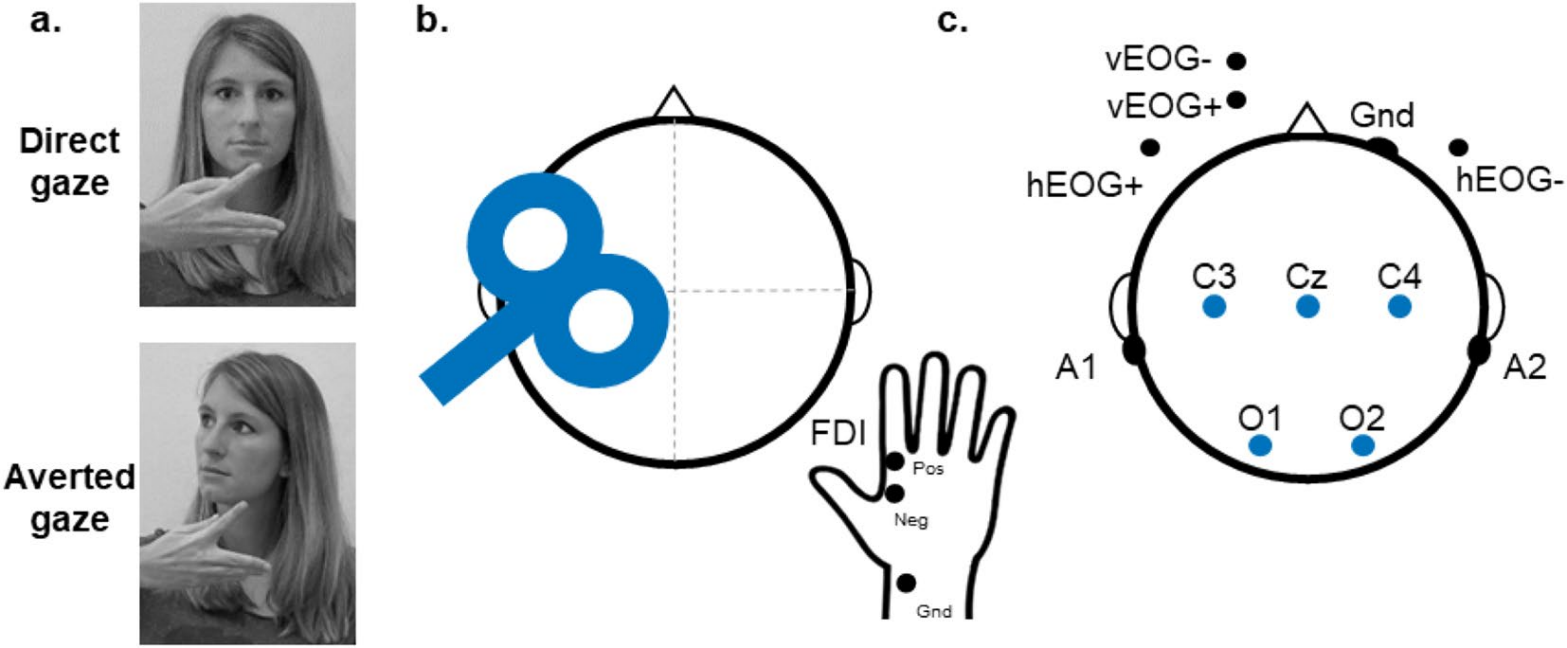
Experimental set-up. (**a**) Illustration of the experimental stimuli, showing a live stimulus person conveying direct or averted gaze while performing a simple finger abduction movement. The stimulus person gives consent to publish this image in an open-access publication of Scientific Reports. (**b**) MEPs induced by TMS over the left primary motor cortex were recorded from EMG electrodes located on the FDI index finger muscle of the right hand. **(c)** Continuous EEG was acquired from electrode sites C3, Cz and C4 to calculate mu suppression, and sites O1 and O2 for alpha suppression.

Participants were seated at a distance of approximately 80 cm from a 20 × 30 cm voltage-sensitive liquid crystal (LC) shutter screen (DreamGlass Group, Spain) attached to a black frame. A ‘live’ female stimulus person (experimenter J.P.) was seated behind the panel (similar set-up as in ref.^31^). During the experimental conditions, the stimulus person’s face was presented through the LC shutter screen for 4 s. Importantly, the stimulus person was unknown to the participants and only briefly interacted with them before the experimental procedure. While the LC screen was transparent, the stimulus person either gazed directly towards the observing participant (i.e. engaging in mutual eye contact) or displayed a gaze 30° to the right (i.e. showing averted gaze). During both gaze conditions, the stimulus person held her right hand horizontally beneath her face with the dorsal side directed to the participants and performed a simple index finger abduction movement (similar movement cue as in ref.^31^). This simple movement allows to record isolated MEPs from the implicated index finger muscle during TMS and is not impacted by (object) familiarity when observed by the participants. The stimulus person bore a neutral expression and tried to avoid eye blinks during the duration of the trial. An illustration of the experimental conditions is provided in Fig. 1A.

Each gaze condition was presented 20 times in 4-s trials with an inter-stimulus-interval of 2 s, during which the shutter remained opaque. The same stimulus protocol was adopted for the TMS and EEG assessment. Participants were instructed to observe and pay close attention to the presented stimuli. In order to ensure that all participants viewed and attended the stimuli properly, participants were asked once at a random point in time during each assessment session to verbally report the stimulus that was presented in the previous trial. Participants were able to correctly report the presented stimulus in 98.5% of the assessments, indicating that they attended the stimuli properly.

### Neurophysiological assessment

#### TMS and EMG recordings

The TMS and EMG electrode set-up is illustrated in Fig. 1B. During observation of the stimuli, single-pulse TMS was administered over M1 with a hand-held 70 mm figure-of-eight coil (oriented approximately 45° relative to the mid-sagittal line) and a Magstim-200 stimulator (Magstim Company Ltd., UK). Optimal coil location for the experimental TMS-stimulation was determined as the site that produced maximal responses (i.e. MEPs) while at rest (“hotspotting”) in the contralateral first dorsal interosseous (FDI) muscle, a muscle implicated in the to-be-observed index finger opening movement. MEPs were obtained by means of surface electromyography (EMG) recordings using disposable adhesive electrodes arranged in a tendon-belly montage. The EMG signal was sampled at 2 kHz, band-pass filtered (5–1000 Hz) and analyzed offline. Resting motor thresholds (rMT) were individually defined as the lowest stimulation intensity that produced a peak-to-peak MEP of at least 50 µV in five out of ten consecutive trials^42^. Experimental stimulation intensity was set at a supra-threshold of 130% of the subject’s rMT.

In each trial, a single magnetic pulse was delivered time-locked to the third second of stimulus presentation (i.e. opening of the LC window). The stimulus person (experimenter J.P.) was trained to coincide the execution of the index finger opening movement with the delivery of the TMS pulse (similar procedure as in ref.^31^). Signal software (version 6.02, Cambridge Electronic Design, UK) and a CED Power 1401 analog-to-digital converting unit (Cambridge Electronic Design, UK) were used for EMG-recordings, triggering of the TMS-stimulator and shifting of the LC window from an opaque to transparent state.

#### EEG data acquisition

The NeXus-32 multimodal acquisition system and BioTrace + software (version 2015a, Mind Media, The Netherlands) were used to collect electroencephalography (EEG) recordings. Continuous EEG was recorded with a cap with 22 sintered Ag/AgCL embedded electrodes (MediFactory, The Netherlands), incorporating 19 EEG channels configured according to the international 10–20 system of electrode placement, two reference electrodes located on the left and right mastoid bones behind the ear (A1 and A2), and an AFz ground electrode. The EEG signal was amplified using a unipolar amplifier. Gentle skin abrasion and electrode paste (combination of electrolytic NuPrep gel and conductive 10–20 paste) were used to reduce electrode impedances below 10 kΩ. Eye movements as well as eye blinks were monitored using two pairs of bipolar electro-oculogram (EOG) electrodes, one pair attached to the external canthi of each eye (horizontal eye movements; hEOG) and one pair attached below and above the left eye (vertical eye movements; vEOG) (Fig. 1C). The sampling rate of the recordings was 256 Hz. E-Prime 2.0 software (Psychology Software Tools Inc., USA) and the NeXus Trigger Interface (NTI, 2048 Hz sample rate; Mind Media, The Netherlands) were used to synchronize stimulus events with the NeXus-32 EEG recordings and the triggering of the LC window.

### Data handling and preprocessing

#### TMS-induced MEPs

Based on the recorded EMG data, peak-to-peak amplitudes of the TMS-induced MEPs were determined using in-house MATLAB scripts (version R2015a, MathWorks Inc., USA). Additionally, background EMG was quantified by calculating the root mean square (RMS) across the 110 to 10 ms interval prior to TMS-stimulation. For a given subject, trials with excessive pre-TMS tonic muscle activity (background EMG exceeding 2.5 standard deviations from the mean) were excluded from analysis. Trials with extreme MEP-amplitudes (exceeding 1.5 interquartile distances from the mean) were also discarded. On average, 17 averted gaze trials (range: 14 – 20) and 18 direct gaze trials (range: 16–20) were retained after this procedure. MEP peak-to-peak amplitudes were log-transformed to conform to normality.

#### EEG mu/alpha suppression calculation

Two participants were excluded from the final analysis due to technical malfunctions of the NeXus Trigger Interface, used for time-locking EEG data with the stimulus presentation. EEG data of the remaining participants (*n* = 30) were preprocessed and analyzed offline using BrainVision Analyzer 2 software (version 2.2, Brain Products GmbH, Germany). The raw EEG signal was mathematically referenced offline to averaged mastoids and filtered using a 0.5–40 Hz IIR band-pass filter with zero phase shift (Butterworth, 24 dB). Taking into account the vEOG and hEOG channels, deflections resulting from eye blinks and horizontal eye movements were removed by the implemented ‘Ocular Correction’ Independent Component Analysis (ICA) module in BrainVision Analyzer 2^43^. Determination of ocular ICA components was based on objective selection criteria implemented in BrainVision Analyzer 2 (i.e. sum of squared correlations with vEOG /hEOG channels > 30%) followed by a visual inspection of the time courses as well as topographies of these components.

Cleaned EEG data were segmented separately for each condition into segments of 4 s, corresponding to the duration of the trial. Segments with residual artifacts exceeding ± 100 µV in amplitude were rejected. Note that one participant was removed from the final analyses due to excessive artifacts (i.e. less than 50% artifact free trials). On average, there were 19 artifact-free trials (range: 17 – 20) in the averted gaze condition and 19 artifact-free trials (range: 14–20) in the direct gaze condition after preprocessing for the remaining 29 participants. For each segment, the spectral power (µV^2^) in the 8–13 Hz range was computed using the Fast Fourier Transform (FFT; including a Hanning window with an attenuation domain of 25%). Obtained power values were then averaged separately for each experimental gaze condition and electrode.

Suppression indices were computed at three central sites (C3, Cz and C4) located over the sensorimotor strip where mu rhythm modulations are expected. To assess the spatial specificity of the gaze-dependent modulations in the mu rhythm, alpha suppression indices were also calculated for occipital electrodes O1 and O2 (Fig. 1C). Mu and alpha suppression indices for each electrode were calculated as the log-transformed ratio of the 8–13 Hz band power during the 4-s trials relative to the power of a 1-s interval prior to the start of the trial (baseline). Log ratios lower than zero indicate suppression.

In order to validate the choice of the 8–13 Hz band and explore the timing of mu suppression, we also performed a continuous wavelet analysis (time interval: 1 s before to 4 s after trial onset, frequency range: 1–20 Hz). As visualized in Supplementary Figure S1, the wavelet analysis confirmed that mu suppression was maximal within the 8–13 Hz frequency range and was evident for approximately the entire duration of the trial (albeit note a small delay in suppression onset at around 0.1 s after trial start).

### Data analysis and statistics

In order to investigate eye contact-induced changes in corticospinal excitability, a mixed-model analysis of variance (ANOVA) with within-subject factor ‘observed gaze’ (averted, direct) was performed on the MEP peak-to-peak amplitude data. The between-subject categorical factor ‘session’ was included as an effect-of-no-interest to account for potential effects of counter-balancing the two assessment sessions (i.e. TMS or EEG first). For the EEG data; it was first tested whether all movement observation conditions elicited a significant suppression relative to the pre-trial baseline segments (as recommended by^22^); i.e. ratio values were tested using single-sample *t*-tests against a value of 0, separately for each gaze condition (Table 1). The mu and alpha suppression indices were analyzed separately using a mixed-model ANOVA with the within-subject factors ‘observed gaze’ (averted, direct) and ‘electrode’ (mu: C3, Cz and C4; alpha: O1 and O2), and the between-subjects factor ‘session’. The partial Eta square (*η*_*p*_^2^) value was calculated as an estimate of effect size.

In order to directly investigate the relationship between the effects of eye contact on the different measures, the ‘eye contact effect’ was quantified for each subject as the percentage change (%change) in the direct gaze condition relative to the averted gaze condition (similar approach as in ref.^44^):

1 $${\text{\% change}} = \left[ {\frac{{response _{direct\,gaze} - {\text{response}} _{averted\,gaze} }}{{{\text{response}} _{averted\,gaze} }}} \right] \times 100$$

Higher mirror responses during the direct versus averted gaze condition are indicated by a positive %change score for MEPs and for mu and alpha suppression indices. Pearson correlation analyses were performed to assess the association between the measures. For all performed correlations, the Cook’s distance metric (*D*) was used to identify influential data points (defined as Cook’s *D* > 1), but none were detected. Here, the coefficient of determination (*R*^2^) is reported as an estimate of effect size. All statistics were calculated with Statistica 10 (StatSoft, USA). Results were considered significant when *p* < 0.05.

## Results

### TMS results

**Figure 2:**
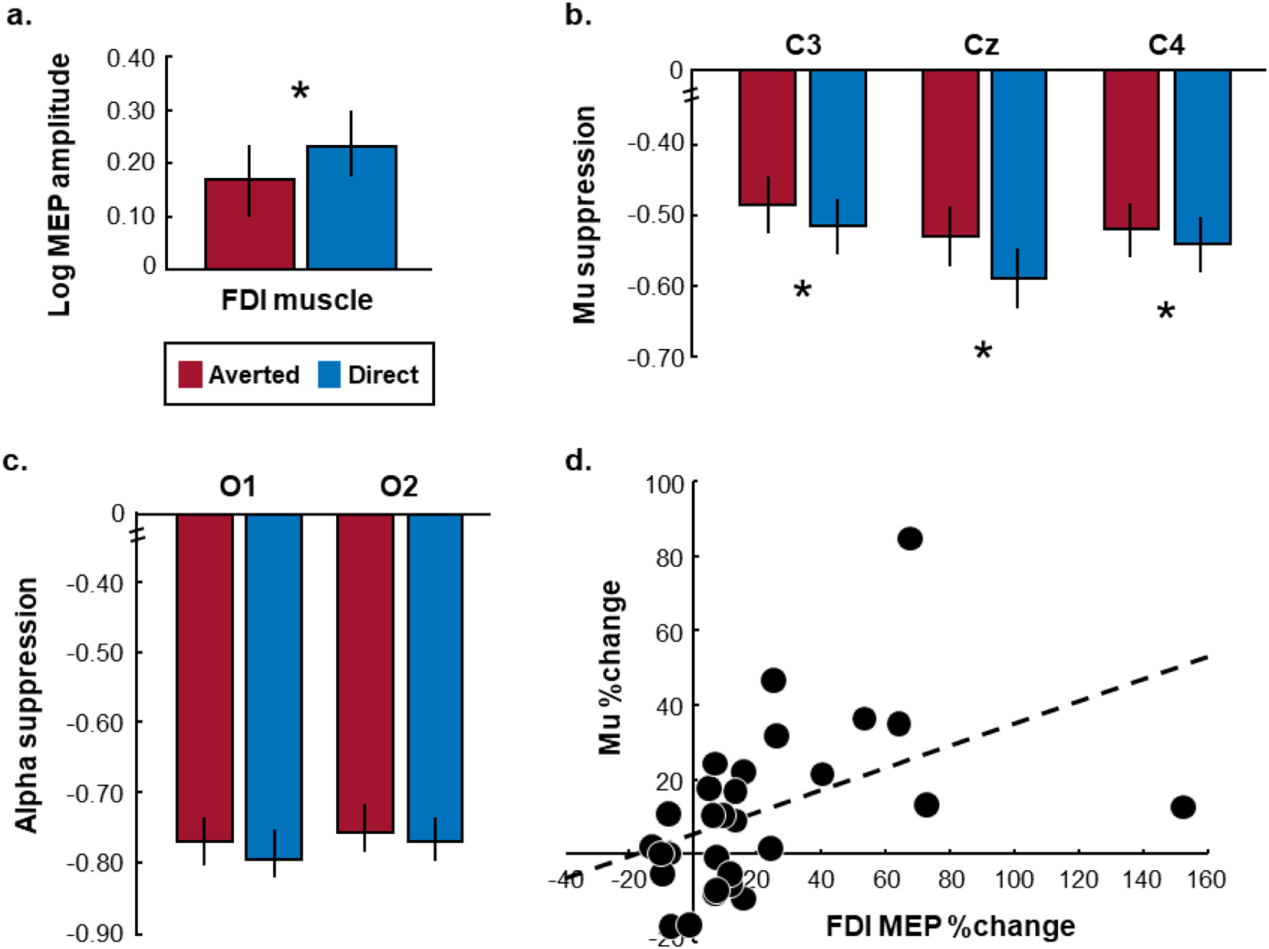
MEP and mu/alpha suppression results. (**a**) Significant effect of perceived eye gaze (direct, averted) on log-transformed MEP peak-to-peak amplitude scores recorded from the FDI muscle (**b**) and mu suppression indices per central electrode**.** *p < .05, vertical error bars denote mean ± SE. (**c**) The effect of perceived eye gaze on alpha suppression over occipital electrodes was not significant. (**d**) A significant positive correlation was found between condition-specific (i.e. eye contact related) changes in MEP amplitude and EEG mu suppression (averaged across central electrodes).

A mixed-model ANOVA with within-subject factor ‘observed gaze’ (averted, direct) and between-subject factor ‘session’ (TMS or EEG first) was performed on the MEP data to investigate the effect of observed gaze on corticospinal excitability. The mean (log-transformed) MEP peak-to-peak amplitudes for each gaze condition is presented in Fig. 2A. In line with our hypothesis, a significant main effect of perceived gaze direction was revealed (*F*(1,30) = 9.22, *p* = 0.005, *η*_*p*_^2^ = 0.24). Thus, in accordance with previous TMS studies^29–31^ investigating the effect of eye-to-eye contact on corticospinal excitability, MEPs recorded from the FDI muscle were significantly higher when movement observation was accompanied with direct gaze from the stimulus person (raw MEP mean: 2.32 mV, SD: 1.97 mV), compared to averted gaze (raw MEP mean: 2.04 mV, SD: 1.79 mV). Note that no significant main or interaction effects were found for the between-subjects ‘session’ factor-of-no-interest, indicating no session-related modulation of the reported effects (all *p* > 0.90).

### EEG results

Significant decreases in mu power with respect to the included rest condition were encountered for each electrode and gaze condition (single sample *t*-tests against 0: all *p* < 0.001; see Table 1), signaling that all gaze conditions induced an overall significant suppression of the mu rhythm during movement observation in the central electrodes.

**Table 1:** Mean central mu and occipital alpha suppression (in 8–13 Hz frequency band) for each gaze condition and electrode.

	Averted gaze		Direct gaze	
	Mean (SD)	*t* against 0	Mean (SD)	*t* against 0
**Central electrodes**				
C3	− 0.66 (0.13)	− 26.71**	− 0.68 (0.11)	− 33.98**
Cz	− 0.71 (0.13)	− 29.78**	− 0.76 (0.13)	− 30.33**
C4	− 0.69 (0.12)	− 30.69**	− 0.71 (0.09)	− 42.02**
**Occipital electrodes**				
O1	− 0.76 (0.18)	− 22.91**	− 0.78 (0.18)	− 23.32**
O2	− 0.74 (0.18)	− 22.32**	− 0.75 (0.19)	− 21.19**

**p < 0.001.

A mixed-model ANOVA with ‘observed gaze’ (averted, direct) and ‘electrode’ (C3, Cz, C4) as within-subject factors and the between-subject factor ‘session’ (TMS or EEG first) revealed a significant main effect of observed eye gaze (*F*(1,26) = 6.97, *p* = 0.01, *η*^2^ = 0.21), but no gaze × electrode interaction (*F*(2,52) = 2.72, *p* = 0.07, *η*_*p*_^2^ = 0.09). This shows that, irrespective of electrode, mu rhythm suppression upon movement observation was more pronounced during the direct versus the averted gaze condition (see Fig. 2B). Also a significant main effect of electrode was revealed (*F*(2,52) = 6.81, *p* = 0.002, *η*_*p*_^2^ = 0.21), indicating that, irrespective of gaze condition, mu rhythm suppression was overall more pronounced in electrode Cz (see Fig. 2B and Table 1). Note that no significant main or interaction effects were found for the between-subjects ‘session’ factor-of-no-interest, indicating no session-related modulation of the reported effects (all *p* > 0.08).

Alpha activity from occipital electrodes O1 and O2 was also significantly suppressed during all gaze conditions compared to rest (all *p* < 0.001; see Table 1). Importantly however, a comparable ANOVA as described above did not indicate a significant main effect of observed eye gaze (*F*(1,25) = 1.90, *p* = 0.18, *η*_*p*_^2^ = 0.07) or electrode (*F*(1,25) = 1.53, *p* = 0.23, *η*_*p*_^2^ = 0.06) for these occipital electrodes, nor an electrode × gaze interaction (*F*(1,25) = 0.08, *p* = 0.78,, *η*_*p*_^2^ = 0.003) (Fig. 2C). During movement observation, occipital alpha suppression was thus not significantly modulated by the observed gaze cues.

### TMS-EEG correlations

Exploration of a potential relationship between FDI MEP amplitudes and mu suppression scores over the central electrodes (averaged score) revealed no significant correlation between absolute MEPs and mu suppression indices for either the direct gaze condition (*r*(29) = -0.21, *p* = 0.26, *R*^2^ = 0.05), nor for the averted gaze condition (*r*(29) = -0.19, *p* = 0.32, *R*^2^ = 0.04). Note that the absence of a significant association persisted when only mu suppression over electrode C3 (contralateral to right-hand MEPs and corresponding to the site of TMS stimulation) was considered (averted gaze: *r*(28) = -0.07, *p* = 0.73, *R*^2^ = 0.005; direct gaze: *r*(28) = 0.01, *p* = 0.95, *R*^2^ < 0.01). Also Bayesian correlation analyses (performed in JASP, version 0.13, stretched beta prior width: 1) further confirmed the absence of a linear association between absolute MEP amplitudes and mu suppression indices (averted gaze: Bayes factor (BF) = 0.37, 95% CI = [-0.50, 0.18]; direct gaze: BF = 0.42, 95% CI = [-0.52, 0.16]).

Interestingly however, it was shown that for the experimental ‘eye contact effect’ (see **Eq. (****1****)**, Method and Materials), 20% of the variance was shared between the TMS and EEG measures, indicating that increments in MEP amplitude in response to direct gaze were significantly associated with similar enhancements of mu suppression (*r*(29) = 0.45, *p* = 0.01, *R*^2^ = 0.20; Fig. 2D). Importantly, this association was specific to the central electrodes, as MEPs were not significantly correlated to alpha suppression indices over occipital electrodes, either in terms of absolute responses (averted gaze: *r*(29) = -0.13, *p* = 0.49, *R*^2^ = 0.02; direct gaze: *r*(29) = -0.15, *p* = 0.43, *R*^2^ = 0.02), or in terms of the experimental ‘eye contact effect’ (*r*(29) = 0.15, *p* = 0.43, *R*^2^ = 0.02). A similar Bayesian correlation analysis as described above confirmed this pattern of results (averted gaze: BF = 0.29, 95% CI = [-0.46, 0.23]; direct gaze: BF = 0.31, 95% CI = [-0.47, 0.22]; ‘eye contact effect’: BF = 0.31, 95% CI = [-0.22, 0.47]).

## Discussion

This study aimed to investigate the impact of observed gaze cues on TMS- and EEG-based measures of mirror system activity. In agreement with previous studies^29–31^, we showed that corticospinal excitability assessed as TMS-induced MEPs upon movement observation was significantly impacted by observed gaze direction from the live stimulus person. Furthermore, we demonstrated that also EEG-based mu suppression in the 8–13 Hz frequency band over the sensorimotor strip (electrodes C3, Cz, C4) was enhanced when observing direct, compared to averted eye gaze from the actor. Importantly, while absolute MEP and mu suppression scores were not related, a significant association was identified between gaze-related changes in corticospinal excitability and mu suppression.

### Social modulation of mirror system activity

The observation that both TMS-induced MEPs and EEG-based mu suppression are modulated by observed gaze cues is in line with the recent notion that motor resonance is not a static process, but is adapted depending on the social context in which the observed movements are embedded. Indeed, other TMS studies have indicated that corticospinal excitability is flexibly modulated by a multitude of social factors, such as, amongst others, emotional body language of the actor^45^, social reciprocity^46^, and the level of observed social interaction^23,47^. Similarly, EEG activity in the mu frequency range has been demonstrated to depend on the extent by which participants are engaged in a social game^48^, the perception of social information such as intentions and emotions^49^ and empathic processes^25^. According to recent theoretical proposals, this subtle control of motor resonance according to the social demands of the environment is proposed to originate in a top-down influence of the mentalizing system^50–52^ and forms an essential competence of humans for flexibly engaging in interpersonal social interactions^50^.

### Association between TMS-induced MEPs and EEG mu rhythm suppression

A second objective of the current research was to further disentangle the relationship between TMS- and EEG-based measures of mirror system functioning at the inter-individual subject level, as previous studies provided an unclear pattern of results^33–36^. In terms of absolute responses, we were unable to establish an association between these two measures. This is in accordance with several previous studies who have directly compared mu suppression and corticospinal excitability in healthy adult participants, either by adopting passive observation of simple hand actions^33^ or goal-directed grasping movements^35^. One additional study, incorporating a mentalizing task to infer others’ intentions in adults with and without ASD, also failed to demonstrate a relationship between these measures^34^.

On the one hand, this lack of a significant association between absolute mu suppression scores and MEPs may relate to the substantial differences in neurophysiological underpinnings and temporo-spatial properties between these measures (i.e. induced activation of a small population of M1 neurons recorded at the peripheral muscles at a discrete time point versus summed post-synaptic electrical activity from a broad population of sensorimotor neurons over a relatively long time period). Although both techniques have been shown to reliably capture mirror system activation (see reviews ^8,22^), it has been suggested that—considering these substantial differences in neurophysiological underpinnings—both techniques might target different aspects of the mirror system.

In this respect, the neural processes triggered by action observation have previously been proposed to be layered in several hierarchically organized functional levels^53,54^. These proposed levels are (i) the muscular level (decoding the pattern of muscle activity necessary to perform the action); (ii) the kinematic level (mapping the effector movement in time and space); (iii) the aim level (including transitive or intransitive short-term goals); and (iv) the intention level (regarding the long-term purpose of the action). Without explicitly framing their design or results in this theoretical structure, Cole et al. (2018) demonstrated that higher mu suppression was associated with superior mentalizing performances, whereas TMS-induced MEPs showed no differences associated with mentalizing^34^. These findings might suggest that the EEG mu rhythm is able to capture higher-order processes such as intentions, but MEPs are not. Note however that the authors opted to deliver the TMS pulse *after* the completion of the video clips conveying the intentions of the actor (i.e. not taking the strict temporal coupling for corticospinal excitability into account). In contrast, another study that investigated mirror system activation across different hierarchical levels (i.e. during observation of intransitive, transitive and interacting hand movements) in adults with and without schizophrenia did reveal a positive association between absolute mu suppression and corticospinal exitability, but only when averaged across *all* conditions depicting biological movement (and participant groups)^36^. In sum, future work is necessary to obtain further information with respect to this hierarchical organization in terms of absolute responses.

Interestingly, while no direct associations were evident between absolute mu suppression scores and MEPs, it was shown that direct gaze-induced increments in MEP amplitude were paralleled by similar enhancements of mu suppression, as indicated by a significant positive relationship of moderate strength between the ‘eye contact effect’ in the EEG and TMS measures. This relationship between gaze-related changes in both measures is an important finding, since it provides initial evidence that the two methods do capture similar flexible changes of these underlying neural processes in response to condition-specific manipulations or contexts (e.g. such as the presentation of socio-communicative cues). In line with the aforementioned theoretical proposals^50–52^, these flexible changes across neurophysiological markers can be considered to reflect a similar “gating” mechanism according to the social saliency or relevance of the observed stimuli, whereby the processing of irrelevant stimuli may be inhibited in order to better process relevant stimuli (see also refs.^24,55^). As such, while the neural correlates underlying absolute MEP and mu suppression scores may differ, it appears that the neural regions involved in processing gaze related cues, i.e. the superior temporal sulcus^56^ or associated regions of the mentalizing network^57^, may exert a similar modulating impact on the (distinct) neurophysiological substrates that drive mu suppression or TMS-induced MEPs upon movement observation.

### Mu rhythm considerations

There are several considerations to be taken into account when evaluating the EEG mu rhythm. For an in-depth discussion, the interested reader is referred to recent reviews of the field^22,58^. Here, we briefly touch upon some relevant issues that motivated our adopted design. First, given the fact that the mu and alpha rhythms oscillate in the same frequency band and show similar response properties^22,59^, we also inspected alpha suppression at the occipital electrodes (O1 and O2). Significant alpha suppression was present during movement observation, suggesting that an attentional component might have been at play during the observation of the different stimuli (see also ref.^48^). It is however important to note that, in contrast to the central mu rhythm, the occipital alpha rhythm was not subjected to gaze-related modulations (i.e. alpha suppression was not significantly stronger during direct vs. averted gaze at occipital electrodes). Furthermore, only eye-contact induced changes in mu suppression indices, but not alpha suppression indices, were significantly associated with eye-contact induced changes in MEPs. In this respect, we believe that these observations highlight the specificity of the mu rhythm in reflecting action-specific mirroring processes, as opposed to reflecting contamination or volume conduction from attentional processes at occipital sites. In line with this notion, a recent study showed that while both central mu and occipital alpha rhythms are indeed similarly suppressed during movement observation, phase synchrony was only evident between central-occipital areas, but not between neighboring occipital-parietal and central-parietal electrodes^60^. These results provide further evidence against a general spread of occipital alpha activity due to volume conduction, but also indicate that visuospatial attention (indexed by occipital alpha) and sensorimotor mirroring (indexed by central mu) are functionally distinct but highly coordinated processes during action observation^16,60^.

Secondly, as the key design feature of mu suppression studies is the comparison of an experimental condition to a baseline condition in which one would not expect mirror system activity, the choice of baseline condition has a substantial impact. Ideally, one collects a baseline period just prior to the period of interest (i.e. the onset of movement), that is identical to the experimental condition, except for this event of interest^61,62^. However, the associative property of the mirror system might pose difficulties for establishing an optimal baseline condition (note that this is not limited to EEG, but also applies to other modalities in action observation research). Although theoretically speaking mirror system activity would be greatest during movement observation, the mere presence of an interactive agent (or object) may elicit early anticipatory reactivity, especially in a design with multiple repetitions^58^. Indeed, studies have demonstrated anticipatory mu suppression prior to action observation^63,64^. As few studies to date have taken advantage of the superior temporal resolution of EEG to examine the temporal dynamics of mu suppression, it is important to take into account that changes in mu might take place before, during or after observation of an action^16^. The additional continuous wavelet analysis (reported in Supplementary Figure S1) supports this notion, by showing that mu suppression was evident for approximately the entire duration of the trial; only a small delay of approximately 0.1 s after trial onset was noted.

Finally, in terms of the spatial domain, it has been suggested that sensorimotor suppression is not only restricted to the central electrodes, but – when employing an EEG cap with minimum 32 electrode channels – can also be observed over nearby premotor (FC electrodes) and somatosensory (CP electrodes) areas^65^. Since the current study employed an EEG cap with only 21 electrodes, it would be of interest for future research to further investigate how mu suppression is spatially distributed over the scalp.

### Limitations

The limitations of this study should be acknowledged. Although we included a verbal report to confirm that participants were attending the stimuli properly, we did not include an online measure of visual attention. Using a similar set-up in combination with eye tracking technology, previous studies from our lab have confirmed that (i) participants look significantly more towards the stimulus person’s eye region during direct gaze trials (i.e. indicative of eye-to-eye contact) and (ii) that participants equally attend the hand area in both gaze conditions (i.e. rendering it unlikely that a shift in attention away from the stimulus space during averted gaze trials underlie the encountered effects)^29–31^. Nevertheless, future studies could benefit from the inclusion of an online measure of visual attention to provide a multifaceted investigation of dyadic eye contact processing.

Secondly, similar to the study by Lapenta et al.^35^, the current study assessed TMS and EEG-related mirror system activity within two separate sessions, whereas the majority of previous studies have measured TMS and EEG simultaneously^33,34,36^. While concurrent recordings may allow for a more direct comparison between both indices, the application of magnetic pulses during TMS induces artifacts in the simultaneously recorded EEG signals (even when TMS-compatible EEG equipment is used). It is therefore necessary to specifically exclude the time window that overlaps with the deliverance of the TMS pulse, which is preferably optimized for the action observation scene. Without adequate adjustments to the experimental design and/or stimuli (as exampled in ref.^33^), some crucial time windows for eliciting mu suppression may need to be removed. Note however that, in terms of absolute values, neither studies adopting separate sessions nor studies using concurrent TMS-EEG measurements were able to demonstrate a robust association between TMS-induced MEPs and the EEG mu rhythm.

Lastly, related to the design choice to enhance the ecological validity of the experiment, it is acknowledged that the employed live set-up might have induced some variability with regard to the exact timing of the movement onset (i.e. at the millisecond level). Although the time course of TMS-assessed corticospinal excitability has been shown to tightly follow the different phases of the observed actions^12,13^, variability depending on the timing of the observed movement was expected to be minimal, since the adopted index finger abduction movement contained only one action phase. Moreover, direct and averted gaze trials were randomized, hence temporal variations were anticipated to occur randomly across trials and participants. A more fine-grained temporal specificity may however be particularly crucial for research studying more complex movements that involve a sequence of distinct action phases (e.g. grasping actions).

## Conclusion

To conclude, both TMS-induced MEPs and EEG-based mu rhythm suppression upon movement observation have independently shown that mirror system activity is significantly impacted by eye contact between observer and performer. Furthermore, this is the first study to date to show that condition-induced (i.e. eye contact-related) changes in corticospinal excitability and mu suppression are related, providing first evidence for a similar gating mechanism that may drive these distinct markers of mirror system functioning.

## Supplementary information

Supplementary Information.
