# Supplementary material for: Enhanced mirroring upon mutual gaze: multimodal evidence from TMS-assessed corticospinal excitability and the EEG mu rhythm
Source: Sci Rep. 2020 Nov 24;10:20449. doi: 10.1038/s41598-020-77508-x (PMC7687883; doi:10.1038/s41598-020-77508-x)
Supplement: Supplementary file 1 — Supplementary Information. [file 41598_2020_77508_MOESM1_ESM.pdf]

Supplementary Information

Enhanced mirroring upon mutual gaze: Multimodal evidence from TMS-assessed corticospinal excitability and the EEG mu rhythm

Jellina Prinsen & Kaat Alaerts

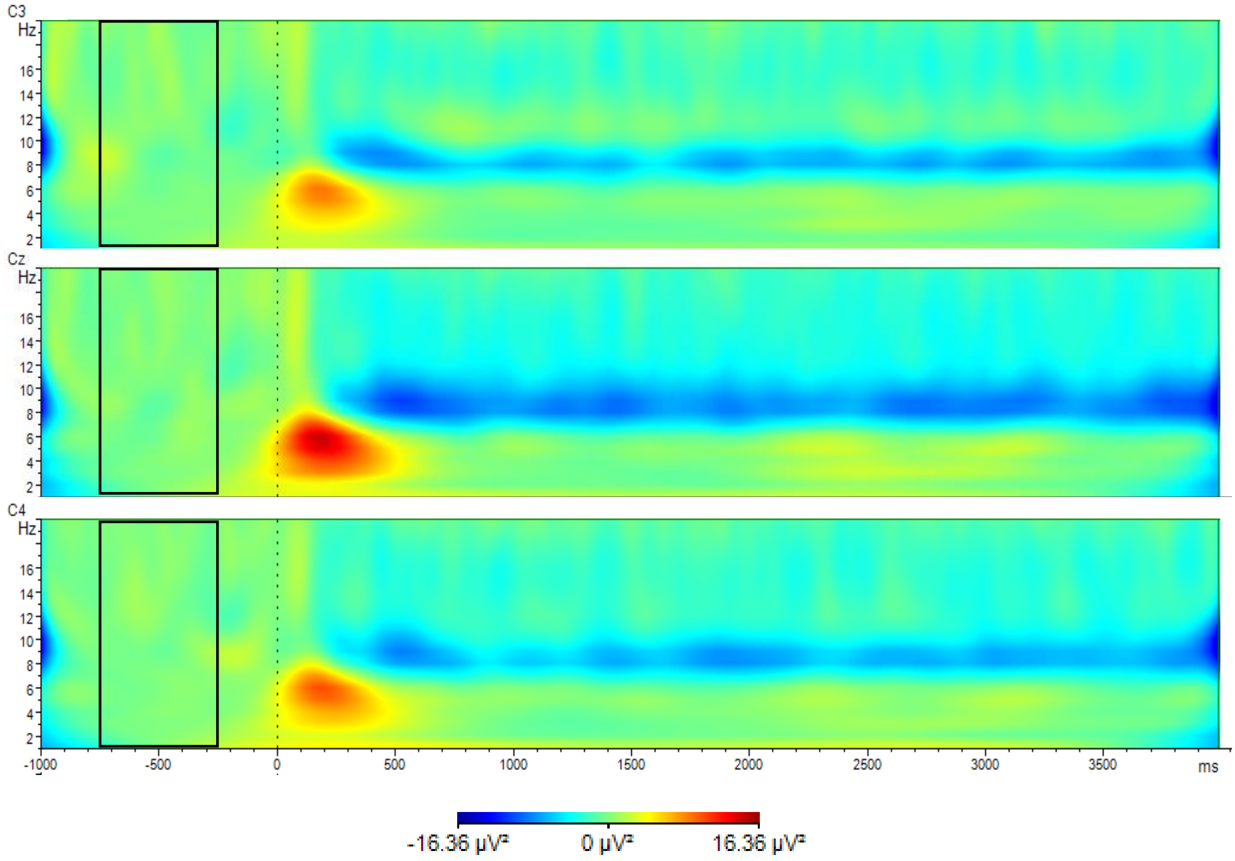

Supplementary Figure S1. Time-frequency power spectrum plot. Spectral power (µV²) for each time-frequency point in the 1–20 Hz range was decomposed using a complex Morlet wavelet (central frequency = 10 Hz, 20 linear steps, Morlet parameter = 5), and computed relative to the mean spectral power in the -750 ms to -250 ms pre-stimulus interval (highlighted within the black rectangles). Illustrates, across conditions and at group level, the average power spectrum for the central electrodes.
